# Metabolome profile and microbial community structure of *Cenchrus fungigraminus* silage under different moisture gradients

**DOI:** 10.3389/fbioe.2025.1657657

**Published:** 2025-09-15

**Authors:** Xiaohan Hou, Xiaohui Chu, Yang Yang, Xue Xiao, Qiongmei Niu, Guilian Shan

**Affiliations:** ^1^ Faculty of Animal Science and Technology Yunnan Agricultural University, Kunming, China; ^2^ Key Laboratory of Animal Nutrition and Feed Science of Yunnan Province, Kunming, China

**Keywords:** *Cenchrus fungigraminus*, moisture content, fermentation quality, bacterial, metabolic pathway

## Abstract

Due to its high biomass yield, *Cenchrus fungigraminus* is widely cultivated in Yunnan. Local herders often adjust the moisture content of fresh grass by sun-drying to improve its suitability for silage. However, the spatiotemporal dynamics of microbial communities and metabolites during its fermentation process remain unclear. In this study, fresh grass was subjected to natural sun-drying for 0, 12, 24, and 36 h, achieving moisture contents of approximately 88%, 77%, 66%, and 50%, respectively, before ensiling. We combined 16S rRNA high-throughput sequencing and LC–MS-based metabolomics to investigate changes in microbial diversity, community structure, and metabolic pathways under different moisture conditions. The results showed that, with prolonged sun exposure, the water-soluble carbohydrate (WSC) and ether extract (EE) of the raw material decreased, and the acid-insoluble fiber (ADF) also decreased; the richness and diversity of the microbial community in the low-moisture group (50%) after silage were significantly higher than those in the high-moisture group, accompanied by an increase in harmful bacteria such as *Clostridium*. In contrast, the abundance of lactic acid bacteria (LAB), including *Lactobacillus* and *Weissella*, showed a significant positive correlation with moisture content. Metabolomics analysis showed that essential amino acid-related pathways (aspartic acid and glutamic acid) were upregulated, while phenolic acid metabolism (protocatechuic acid and gallic acid) was downregulated, reflecting the differential regulation of fermentation products under different moisture conditions. In summary, although excessive sun-drying can optimize the fiber structure and palatability of raw materials, overly low moisture levels are unfavorable for the proliferation of probiotics and may compromise silage quality. Maintaining a higher moisture content (≥75%) for silage is more conducive to fermentation stability and nutritional value, providing a theoretical basis for optimizing the silage process of *Cenchrus fungigraminus*.

## 1 Introduction

*Cenchrus fungigraminus* (*C*. *fungigraminus*) is a perennial C_4_ grass plant with the characteristics of rapid growth, high biomass production, and strong adaptability to tropical and subtropical environments (Song et al., 2025). After only 4 weeks of growth, its crude protein content can exceed 10.8% (Liu et al., 2015), and it can be harvested multiple times per year, producing 200–400 t ha^-1^ of fresh forage annually—yields (Aimable et al., 2025) comparable to those of other high-biomass C_4_ species such as *Cenchrus purpureus* and *Pennisetum purpureum* (Li et al., 2024). Despite its clear potential as a ruminant feedstock in southern China, systematic studies on its ensiling behavior and the interplay between moisture regulation, microbial succession, and metabolite production are still lacking.

Ensiling under strictly anaerobic conditions is the most widely used method for forage preservation. However, the moisture content of raw materials exerts a decisive influence on fermentation pathways. Silages are conventionally classified as high-moisture (>75%), wilted (65%–75%), or semi-dry (<65%) based on their water content (Queiroz et al., 2018), although optimal ranges vary with crop type. For example, *Lolium perenne* L., when harvested before flowering, benefits from air-drying to below 50% moisture to promote complete fermentation (Fluck et al., 2018), whereas direct ensiling of high-moisture forages (e.g., Pennisetum hybridum at > 80%) often leads to proteolysis and nutrient losses (A et al., 2019). Fresh *C. fungigraminus* typically contains over 85% moisture, which can induce diarrhea in livestock if fed directly, yet its protein-rich profile underscores its silage potential.

Changes in the epiphytic microbiota driven by moisture determine the spectrum of fermentation metabolites—including organic acids, amino acids, and phenols, which ultimately determine the quality and nutritional value of silage (Kung et al., 2018). To date, no study has combined high-throughput sequencing and untargeted metabolomics to evaluate how controlled drying influences both the microbiome and metabolite landscape of *C. fungigraminus* silage. In this study, we simulate practical pre-ensiling treatments by sun-drying fresh grass for 0, 12, 24, and 36 h to achieve moisture levels corresponding to high-moisture, wilted, and semi-dry silages. We then apply 16S rRNA gene sequencing and LC–MS metabolomics to elucidate the coupled effects of moisture adjustment on fermentation-related microbial dynamics and metabolic profiles, thereby providing a scientific basis for optimizing *C. fungigraminus* silage production.

## 2 Materials and methods

### 2.1 Ensiling of *C. fungigraminus*

On 1 July 2024, *C. fungigraminus* plants (approximately 2.4 m in height) were harvested from the forage production base of Qilin Breeding Cooperative, Xundian County, Yunnan Province, China (25°20′–26°01′N, 102°41′–103°33′E; altitude 1,900 m; subtropical monsoon climate). The apical portions were cut and chopped into 2–3 cm pieces using a Model 932R-100 chopping machine (Kunming Rizhao Hefeng Power Generating Machine Co., Ltd., Kunming, China). Fresh material was transported to the Grass Science Laboratory at Yunnan Agricultural University, where initial moisture content (88.90%) was determined at 18:00 on 1 July 2024. The experiment simulated the drying treatment method used by local farmers to explore the actual production challenges and obtain four moisture gradients. The fresh forage was spread in a well-ventilated outdoor area and subjected to natural sun-drying for 0 h, 12 h, 24 h, and 36 h (ending at 18:00 on 1 July, 08:00 on 2 July, 18:00 on 2 July, and 08:00 on 3 July 2024, respectively). Fresh *C. fungigraminus* plants was turned every 2 h to ensure uniform drying, resulting in target moisture contents of approximately 88%, 77%, 66%, and 50%. Samples from each time point were collected for chemical composition and microbial community analyses (Omicsmart High-throughput Sequencing Platform, Gene Denovo Biotechnology Co., Ltd., Guangzhou, China).

For ensiling, 2 kg of material from each drying treatment was compacted into 5 L high-density polyethylene buckets with lids (Kunming Zhenxi Plastic Products Co., Ltd., Kunming, China), sealed with polyvinyl chloride film (Nanya Plastic Film Co., Ltd., Nantong, China) and stored at ambient temperature for 60 days. The fresh grass was evenly spread and dried on the ground. The five random areas are collected and mixed into one sample to complete the sampling under the treatment. Then, a sample was divided into three parts to achieve three repetitions.

### 2.2 Determination of nutritional quality and chemical indicators

Fresh and ensiled samples were dried at 85 °C for 48 h in a forced-air oven (Model DHG-9240, Shanghai Yiheng Scientific Instrument Co., Ltd., Shanghai, China) to determine moisture and dry matter (DM) content (Zou et al., 2021). Dried material was milled to pass a 1.0-mm screen using a small hay crusher (Model SM-200, Beijing Yishunchen Technology Co., Ltd., Beijing, China).

Neutral detergent fiber (NDF) and acid detergent fiber (ADF) were measured using the Van Soest method (Van Soest et al., 1991) on a fully automated fiber analyzer (Model F2000, Hanon Advanced Technology Group Co., Ltd., Jinan, China). Crude protein (CP) was determined using the Kjeldahl method (Hasan, 2015) on a semi-automatic Kjeldahl nitrogen analyzer (Model K1100, Hanon Advanced Technology Group Co., Ltd., Jinan, China). Ether extract (EE) was quantified using Soxhlet extraction (Model SOX606, Hanon Advanced Technology Group Co., Ltd., Jinan, China). Water-soluble carbohydrates (WSCs) were assayed using the anthrone–sulfuric acid colorimetric method. Ash content was determined by incineration at 550 °C for 6 h in a muffle furnace (Model SX2-4-10, Nabertherm GmbH, Lilienthal, Germany).

For pH and ammonia–nitrogen analyses, 10 g of fresh or ensiled material was mixed with 90 mL of sterile deionized water, shaken for 24 h at 4 °C, and then filtered. pH was measured using a bench-top pH meter (Model SG23, Mettler Toledo, Zurich, Switzerland). Ammonia–nitrogen was determined using the ninhydrin hydrate colorimetric method (Broderick and Kang, 1980).

Organic acids (lactic, acetic, propionic, and butyric acid) were quantified through high-performance liquid chromatography (HPLC, Model LC-20AT, Shimadzu Corporation, Kyoto, Japan). Sample extracts were prepared by diluting 2 g (±0.01 g) of homogenized material to 25 mL with deionized water, filtering through a 0.45 μm membrane (MilliporeSigma, Burlington, United States), and injecting 20 μL. Separation was performed on a Shim-pack C18-AQ Column (150 mm × 4.6 mm, 5 μm; Shimadzu Corporation, Kyoto, Japan) at 30 °C, using 0.1% phosphoric acid and propylene phosphate (3:1, v/v) as the mobile phase at a flow rate of 0.6 mL min^-1^, with detection at 210 nm.

### 2.3 Bacteria composition analysis

Total microbial DNA was extracted from 0.5 g of sample using the HiPure Stool DNA Kit (Magen Biotech Co., Ltd., Guangzhou, China). DNA concentration and purity were assessed using a NanoDrop 2000 Spectrophotometer (Thermo Fisher Scientific Inc., Waltham, Massachusetts, United States), and integrity was assessed by electrophoresis on a 1% agarose gel using a DYY-6C system (Beijing Liuyi Biotechnology Co., Ltd., Beijing, China) and visualized using a Tanon-2500 Gel Documentation System (Tanon Science & Technology Co., Ltd., Shanghai, China).

The V5–V7 region of the bacterial 16S rRNA gene was amplified using primers 799F (5′-AACMGGATTAGATACCCKG-3′) and 1193R (5′-ACG​TCA​TCC​CCC​CAC​CTT​CC-3′) in a Bio-Rad T100 Thermal Cycler (Bio-Rad Laboratories, Hercules, California, United States) under the following conditions: 95 °C for 2 min; 30 cycles of 95 °C for 1 min, 60 °C for 1 min, and 72 °C for 1 min; and a final extension at 72 °C for 7 min. Amplicons were purified with AMPure XP Beads (Beckman Coulter, Brea, California, United States) and quantified using a Qubit 3.0 fluorometer (Thermo Fisher Scientific Inc., Waltham, Massachusetts, United States), and libraries were constructed using the Illumina DNA Prep Kit (Illumina, San Diego, California, United States). Library quality was verified using an ABI StepOnePlus Real-Time PCR System (Applied Biosystems, Foster City, California, United States).

Raw reads were quality-filtered using FASTP version 0.18.0 (Chen et al., 2018), merged using FLASH version 1.2.11 (Magoc and Salzberg, 2011), and processed in QIIME version 1.9.1. Sequences were clustered into operational taxonomic units at 97% similarity using UPARSE version 9.2.64 with chimeras removed via UCHIME. Representative sequences were classified against the SILVA database version 138.2 (Pruesse et al., 2007) using the RDP Classifier version 2.2 (Wang et al., 2007), with a confidence threshold of 0.8 s.

### 2.4 Sequencing and analysis of metabolites

A 100 mg sample was taken in 1 mL of cold 90% methanol. The lysate was homogenized using an MP homogenizer (24 × 2, 6.0M/S, 60 s, twice). The homogenate was sonicated at low temperature (30 min/once, twice). The mixture was centrifuged for 20 min (14,000 g, 4 °C). The supernatant was dried in a vacuum centrifuge. For LC–MS analysis, the samples were re-dissolved in 100 μL acetonitrile/water (1:1, v/v) solvent. The raw MS data were converted to MzXML files using ProteoWizard MSConvert (v3.0.6428) before importing into freely available XCMS software (online 3.7.1). For peak picking, the following parameters were used: centWave m/z = 10 ppm, peakwidth = c (10, 60), and prefilter = c (10, 100). For peak grouping, bw = 5, mzwid = 0.025, and minfrac = 0.5 were used. CAMERA (Collection of Algorithms of MEtabolite pRofile Annotation) was used for the annotation of isotopes and adducts. In the extracted ion features, only the variables having more than 50% of the nonzero measurement values in at least one group were retained. Compound identification of metabolites was performed by comparing accurate m/z values (<10 ppm) and MS/MS spectra with an in-house database established using available authentic standards. The missing data were filled using the K-nearest neighbor (KNN) method, and features with RSD greater than 50% were filtered out.

### 2.5 Statistical analysis

The chemical composition and microorganisms of the raw materials of forage for different drying periods, along with the differences in chemical composition, fermentation quality, and microorganisms of silage forage, were evaluated through ANOVA using SPSS (version 23.0). Heatmap analysis used the “cor ()” function in R to calculate the correlation coefficient, the “cor. test ()” function to perform data verification, and the “corrplot” to perform plotting. P < 0.05 is considered to be a significant difference.

## 3 Results

### 3.1 Effect of drying time on chemical composition and microbial community structure of *C. fungigraminus*

Drying had a dual effect on the nutritional quality of the raw materials (Table 1). It was reflected in the fact that compared with the 88% moisture content (FS). The moisture of 77% (S12), 66% (S24), and 50% (S36) significantly increased the CP and DM contents (*p* < 0.05). This indicates that prolonged natural drying alters the nutrient composition of the raw material: WSC and EE decreased, reducing substrates available for silage fermentation, whereas ADF also decreased, potentially improving feed palatability. This finding was consistent with previous research (Fonnesbeck et al., 1986).

**TABLE 1:** Effect of drying time on chemical composition of fresh grass of *C. fungigraminus*.

Item	FS	S12	S24	S36	SEM	*p*-value
DM g/kg FM	112.9d	235.1c	452.1b	538.2a	42.26	<0.01
CP g/kg DM)	9.555c	10.44b	11.07a	11.11a	0.1574	<0.01
EE g/kg DM	10.1a	7.035b	5.587c	5.017c	0.4925	<0.01
WSC g/kg DM	12.07a	4.194b	4.085b	3.183b	0.898884	<0.01
NDF (g/kg DM	69.69a	70.25a	70.71a	68.10c	0.246164	0.002
ADF g/kg DM	45.73a	44.27b	42.63b	42.14b	3.438693	<0.01

Notes: FS, undried; S12, dried for 12 h; S24, dried for 24 h; S36, dried for 36 h; DM, dry matter; CP, crude protein; EE, ether extract; WSC, water-soluble carbohydrate; NDF, neutral detergent fiber; ADF, acid detergent fiber. Different lowercase letters indicate significant differences among treatments (*p* < 0.05). SEM, standard error of the mean.

**FIGURE 1:**
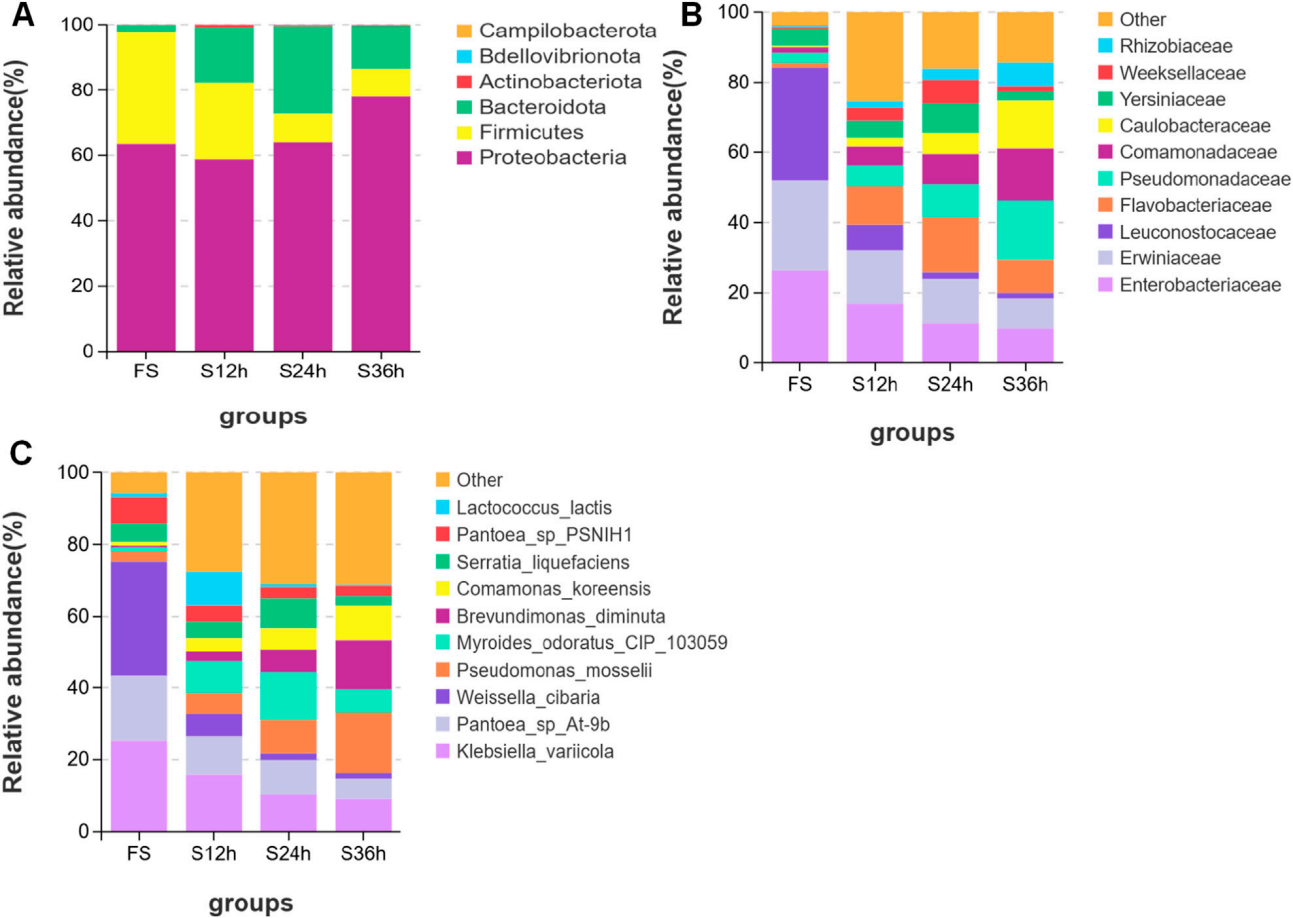
Effect of drying time on bacterial community structure of fresh grass of *Cenchrus fungigraminus*. The species distribution stacking map can visually display the composition and proportion of each group of species, reflecting the changes in species between groups. By comparing and annotating against the database, the obtained OTUs were classified by species, and the distribution of each group of crop species was analyzed at the phylum, class, order, family, genus, and species levels. **(A)** Phylum level. **(B)** Family level. **(C)** Species level. Each processed OTU value was obtained as the average of three replicates.

Drying had a great impact on the microbial community of forage grass raw materials (Figure 1), which was reflected by the fact that, compared with the 88% moisture group (FS), moisture contents of 77% (S12), 66% (S24), and 50% (S36) increased the relative abundance of *Bacteroidetes* and *Proteobacteria*, while the relative abundance of the phylum Firmicutes showed a downward trend with increasing drying time. At the family level, the abundance of Weissella decreased with an increase in the drying time, and the abundance of some anaerobic bacteria increased, such as *Klebsiella*. At the species level, the abundance of the beneficial bacterium *Weissella* decreased dramatically with an increase in the drying time, while the abundance of the harmful bacteria, such as *Klebsiella*, showed a downward trend, but less significantly than *Weissella*. Regarding the effect of drying on the alpha diversity of bacterial communities in the raw materials of *C. fungigraminus* (Table 2), compared with the FS group, the drying treatments (S12, S24, and S36 groups) significantly increased the indices of fresh forage, including Sobs, Shannon, and others. This was plausible due to the proliferation of harmful bacteria during the drying process, which led to an increase in the richness and diversity of bacterial communities. The growth of harmful bacteria significantly reduced the contents of WSC and EE in the fresh material of *C. fungigraminus*.

**TABLE 2:** Effect of drying time on the α-diversity of bacterial communities in the fresh grass of *C. fungigraminus*.

Group	FS	S12	S24	S36	SEM	*p*-Value
Sobs	62b	82.333a	82.333a	81a	4.982	0.12
Shannon	2.803b	4.154a	4.196a	4.044a	0.334	<0.01
Simpson	0.776b	0.91a	0.919a	0.9a	0.034	0.002
Chao	69.567b	87.678ab	94.833a	86.692ab	5.358	0.06
Ace	72.661b	91.269ab	95.026a	90.178ab	4.984	0.09
Goods	0.999a	0.999a	0.999a	0.999a	<0.01	0.825

Notes: Different lowercase letters indicate significant differences among treatments (*p* < 0.05).

### 3.2 Effect of drying time on chemical composition and microbial community structure of silage in *C. fungigraminus*

The moisture content of raw materials strongly influenced both the fermentation quality and nutritional value of *C. fungigraminus* silage (Table 3). It was reflected in the reduction in the lactic acid content in the drying treatment [moisture contents of 77% (S12), 66% (S24), and 50% (S36)] compared with the 88% moisture content, and the pH value was higher than 4.2, failing to meet the standard for high-quality silage. The content of acetic acid increased with the decreased moisture content. In the context of silage fermentation, fluctuations in moisture content predominantly facilitate the proliferation of undesirable microorganisms. These microorganisms consume WSC and EE present in the raw materials. Consequently, it is necessary to rely on heterofermentative lactic acid bacteria, which produce acetic acid, to inhibit the formation of an adverse fermentation environment induced by these undesirable microorganisms (Table 1), resulting in insufficient substrates for silage fermentation and insufficient acid production, which, in turn, causes the proliferation of harmful bacteria in the silage microenvironment. Heterofermentative lactic acid bacteria convert part of the lactic acid into acetic acid to counteract the negative effects of adverse microorganisms, thereby decreasing the lactic acid content, and the pH value of the silage in the drying groups remained at a high level. In comparison, the pH of silage with 88% moisture was 3.51, meeting the standard of excellent silage pH. The contents of propionic acid, butyric acid, and NH_3_–N, due to the low moisture content, in the drying groups increased compared with those of the FS group. The butyric acid content in the 50% moisture group also increased with decreasing moisture content.

**TABLE 3:** Effect of drying time on the fermentation quality and nutritional quality of silage in *C. fungigraminus*.

Item	FS	S12	S24	S36	SEM	*p*-value
pH	3.51c	5.00a	4.84 ab	4.753b	0.148282	<0.01
LA g/kg DM	24.2	13.68	14.9	15.67	3.117358	<0.01
AA g/kg DM	5.76	9.8	10.97	11.16	1.101154	<0.01
PA g/kg DM	-	1.19	2.63	4.46	0.446044	<0.01
BA g/kg DM	0.21	1.05	0.92	1.76	0.3875	<0.01
EE g/kg DM	5.4667b	4.836bc	7.118a	4.266c	0.266865	<0.01
NH3-N μmol/g FM)	16.37b	16.42b	19.85 ab	28.76a	1.264227	0.109
WSC g/kg DM	2.902a	0.944c	0.944c	0.9141b	0.213061	<0.01
CP g/kg DM	9.455b	9.2804b	9.234b	10.62a	0.140509	0.08
NDF g/kg DM	60.14b	65.02a	59.59b	56.2c	0.0062	<0.01
ADF g/kg DM	27.32b	31.09a	27.88b	28.26b	0.0049	<0.01
Ash g/kg DM	8.12b	8.05b	9.29a	9.95a	0.02867	<0.01

Notes: FS, undried; S12, dried for 12 h; S24, dried for 24 h; S36, dry dried for 36 h; LA, lactic acid; AA, acetic acid; PA, propionic acid; BA, butyric acid; EE, ether extract; WSC, water-soluble carbohydrate; CP, crude protein; NDF, neutral detergent fiber; ADF, acid detergent fiber; Ash, crude ash. Different lowercase letters indicate significant differences among treatments (*p* < 0.05). SEM, standard error of the mean.

The EE of silage is generally slightly increased (Merkeviit-Venslov et al., 2022). In this experiment, the EE of the S24 group increased compared with the raw materials, while that in the other treatment groups decreased, which led to complex reasons. The reduction in 88% moisture (FS) may be due to the high moisture content of the raw materials, which caused substantial exudation and increased the risk of EE loss (Smith et al., 2020). The 77% moisture (S12) and 66% moisture groups (S36) may have been affected by mold growth, which consumed a considerable amount of EE, thereby reducing the overall content (Zhou J. et al., 2022). The WSC content of the FS group was 2.9%, while the WSC retention of other treatment groups was less than 1%. The NH_3_–N content in all treatment groups was higher than 10%. When the WSC content was less than 3%, the activity of *Clostridium* was out of control. This may explain why the NH_3_–N content in this test did not meet the high-quality feed standards (Goldsztejn et al., 2020). It showed that the addition of lactic acid bacteria was necessary to quickly acidify and inhibit *Clostridium* activities and ensure the output of high-quality silage.

**FIGURE 2:**
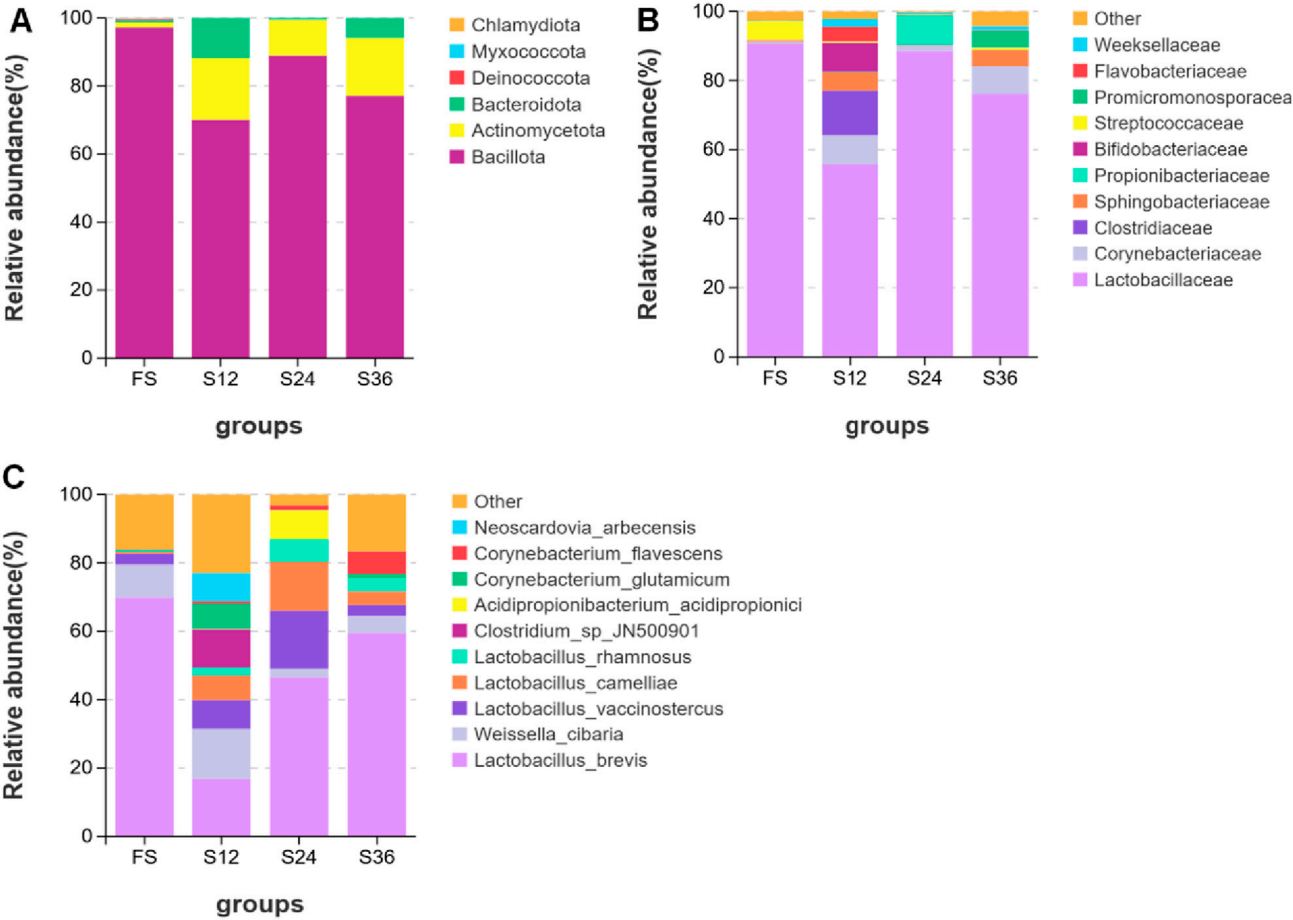
Effect of drying and adjusting different moisture contents on bacterial community structure of silage in *Cenchrus fungigraminus*. The species distribution stacking map can visually display the composition and proportion of each group of species, reflecting the changes in species between groups. By comparing and annotating against the database, the obtained OTUs were classified by species, and the distribution of each group of crop species was analyzed at the phylum, class, order, family, genus, and species levels. **(A)** Phylum level. **(B)** Family level. **(C)** Species level. Each processed OTU was obtained as the average of three replicates.

The drying time had a great impact on the bacterial community structure and diversity of the silage of *C. fungigraminus*. As shown in Figure 2, at the phylum level, the silage in the 88% moisture group (FS) had an absolute advantage with *Bacillus*, and the relative abundance of Actinobacteria [moisture groups of 77% (S12), 66% (S24), and 50% (S36)] was significantly increased compared with that in the 88% moisture group. At the family level, the *Lactobacillus* family had an absolute advantage in 88% moisture and 66% moisture groups. The relative abundance of *Clostridium* in the 77% moisture group was higher than that in the other treatment groups. In addition, the relative abundance of Corynebacterium in 77% moisture and 50% moisture groups was higher than that in the other treatment groups. At the species level, *Lactobacillus brevis* dominated in the 88% moisture group, followed by *Weissella*. Compared with that in the 88% moisture group, the relative abundance of *Lactobacillus brevis* in the 77% moisture group was significantly reduced, and the relative abundance of *Weissella* increased. The diversity of species in the 66% moisture and 50% moisture groups showed an increasing trend. This indicated that the reduction in the moisture content in drying caused changes in the microbial community structure of the silage in *C. fungigraminus*, the abundance of the lactic acid bacteria population increased, and the types and abundance of harmful bacteria would also be increased. From Table 3, the coverage of the processing groups was close to 1, which indicated that the microbial detection evaluation was relatively comprehensive. Compared with the 88% moisture group, the Sobs and Shannon values of the drying groups [moisture groups of 77% (S12), 66% (S24), and 50% (S36)] increased, indicating that the decrease in moisture content increased the microbial diversity during silage fermentation.

### 3.3 Analysis of the KEGG enrichment pathway of different metabolites of silage in *C. fungigraminus* for different drying times

**FIGURE 3:**
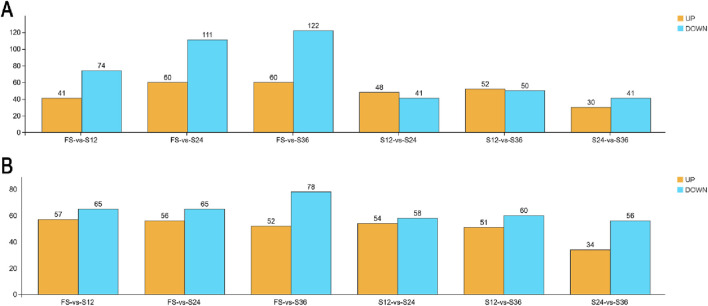
Effect of drying and adjusting moisture contents on differential metabolites of silage in *Cenchrus fungigraminus*. The number of differential genes that meet threshold screening in each comparison group was counted. After threshold screening, individual multiple comparison groups were expressed in different colors to upregulate and downregulate the number of different genes. By default, yellow represents up-adjustment, and blue represents down-adjustment. **(A)** POS (positive ion); **(B)** NEG (negative ion). The test value *p* < 0.05, the difference multiple is 1, and the VIP value is 1.

As the moisture content decreased, the number of differential metabolites increased compared to that in the 88% moisture group (FS), which was related to the changes in the raw material microbial community (Figure 3). Amino acid substances such as lysine and alanine showed an upregulation trend because the microbial activity in the drying groups was more abundant and active than that in the silage microenvironment at 88% moisture (Table 4). Butyric acid showed an upregulation trend, which indicated that the reproduction and fermentation of lactic acid bacteria in the drying groups did not completely develop. The proliferation of harmful bacterial, such as *Clostridium*, promoted an increase in butyric acid production. At the same time, benzoic acid and m-hydroxybenzoic acid also showed an upregulation trend, indicating that the bacterial community favoring acid production in the silage microenvironment exerted a negative impact, providing feedback against harmful bacteria and generating the continuous impact of harmful substances through the production of antibacterial substances (Table 5).

**TABLE 4:** Effect of drying and adjusting different moisture contents on the α-diversity index of the bacterial community of silage in *C. fungigraminus*.

Group	FS	S12	S24	S36	SEM	*p*-value
sobs	34.33c	51.33a	37.33bc	42.33b	20.04	0.004
Shannon	1.79c	3.9a	2.38b	2.45b	0.12	<0.01
Simpson	0.49c	0.9a	0.71b	0.63b	0.01	<0.01
Chao	35.67c	51.81a	40.83bc	45.92ab	20.04	0.017
ace	35.67c	52.9a	41.51bc	45.43ab	19.85	0.01
coverage	0.9997a	0.9997a	0.9994a	0.9995a	0	0.186

Notes: Different lowercase letters indicate significant differences among treatments (*p* < 0.05).

**TABLE 5:** Drying and adjustment of the main differential metabolites of silage under different moisture contents.

Metabolite	FS_vs._S12	FS_vs._S24	FS_vs._S36	S12_vs._S24	S12_vs._S36	S24_vs._S36
Hydrocinnamic acid	Up	Up	Up	Up	Up	Up
L-Aspartic acid	Up	Up	Up	-	-	Up
Benzoic acid	Up	Up	Up	-	-	-
Succinic acid	Up	Up	Up	up	Up	-
2-Methylbenzoic acid	Up	Up	Up	-	-	-
Isovanillic acid	Up	Up	Up	-	-	-
L-Glutamic acid	Up	Up	Up	-	-	Up
3,5-Dihydroxybenzoic acid	Up	Up	Up	-	-	Up
Ascorbic acid	Up	Up	Up	-	-	Up
p-Hydroxyphenylacetic acid	Up	Up	Up	-	Up	-
L-Lysine	Up	Up	Up	-	-	Up
L-Alanine	Up	Up	Up	-	-	-
5-Aminopentanoic acid	Up	Up	Up	-	-	-
trans-Cinnamic acid	Up	Up	Up	Up	Up	-
p-Cresol	Up	Up	Up	-	-	-
Isobutyric acid	Up	Up	Up	-	-	-
Porphobilinogen	Up	Up	Up	-	-	Up
Protocatechuic acid	Down	Down	Down	-	-	-
Gentisic acid	Down	Down	Down	-	-	-
Isocitric acid	Down	Down	Down	-	-	-
Syringic acid	Down	Down	Down	-	-	-
2-Aminoadipic acid	Down	Down	Down	-	-	-
2-Methylene-4-oxopentanedioic acid	Down	Down	Down	-	-	-
Oleic acid	-	-	-	up	Up	-
16-Hydroxyhexadecanoic acid	-	-	-	up	Up	-
Palmitic acid	-	-	-	up	Up	-
Floionolic acid	-	-	-	down	Down	-
2-Isopropylmalic acid	-	-	-	-	-	Up
N2-Acetylornithine	-	-	-	-	-	Up
2-Oxo-4-methylthiobutanoic acid	-	-	-	-	-	Down
4-Hydroxycinnamic acid	-	-	-	-	-	Up
Xanthine	-	-	-	-	-	Up
Hypoxanthine	-	-	-	-	-	Up
D-Threonine	-	-	-	-	-	Up
Pantothenic acid	-	-	-	-	-	Up
4-Acetamidobutanoic acid	-	-	-	-	-	Up
D-2-Hydroxyglutaric acid	-	-	-	-	-	Up
5-Hydroxynicotinic acid	-	-	-	-	-	Up
17a-Hydroxypregnenolone	-	-	-	-	-	Up
3-Methylxanthine	-	-	-	-	-	Up
3-Methylthiopropionic acid	-	-	-	-	-	Up
Jasmonic acid	-	-	-	-	-	Down

**FIGURE 4:**
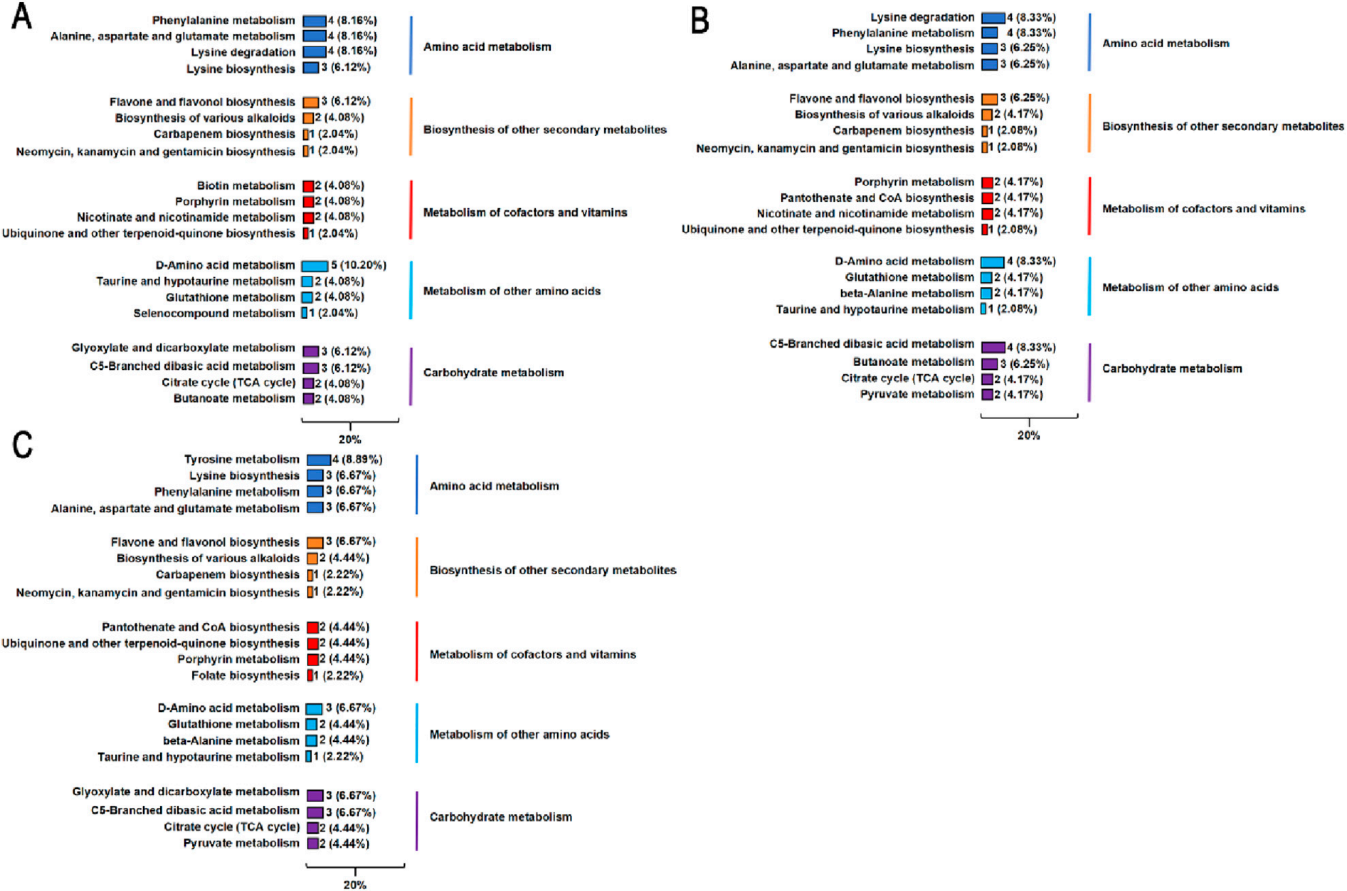
Top five differential metabolite enrichment pathways in silage of *Cenchrus fungigraminus*. The left level represents the path name, and the right level represents the KEGG_B Class information. Band numbers represent the top four differential metabolites under this pathway. The band ratio represents the proportion of differential metabolites under this pathway among the top 20 differential metabolites. **(A)** FS vs. S12, **(B)** FS vs. S24, and **(C)** FS vs. S36.

This experiment selected the top five KEGG enrichment pathways and the top four metabolic pathways under this pathway for analysis (Figure 4). The most common pathway was amino acid metabolism, which is a core link in the dynamic changes of proteins during silage fermentation. In the comparison of 88% moisture with 77%, 66%, and 50%, enrichment of the valine metabolic pathway (lysine-related) and the tyrosine metabolic pathway (phenylephrine-related) was detected. This was in response to the detection of *Clostridium* in the microbial community. Lysine and tyrosine are converted into cadaverine and tyrosine, respectively, through the tyrosine metabolic pathway by *Clostridium*. After feeding ruminants, this could cause negative effects such as hypertension and tissue necrosis. The vitamin cofactor metabolic pathway (Vitam) and carbohydrate metabolic pathway (Carbo) were enriched. In the vitamin cofactor metabolic pathway, nicotinate supports the supply of NAD+, allowing lactic acid to be synthesized normally. Biotin can accelerate the conversion of glucose to lactic acid, and the supply of biotin in the experiment was insufficient. The reason is that the isovaleric acid in metabolites showed an upregulation trend, which indicated that *Clostridium* was competing with lactic acid bacteria for nutrients on valine substrates. Moreover, as the moisture content decreased, the competitiveness of *Clostridium* increased; isovaleric acid had a significant impact on pH, making it impossible for the silage system to effectively inhibit spoilage bacteria, which led to slow growth of lactic acid bacteria and enhanced fermentation of butyric acid. In the carbohydrate metabolism pathway, lactic acid bacteria obtain energy through glycerol phospholipid metabolism, and at the same time, glycerol phospholipid metabolism provides the fermentation substrate for TCA. The TCA cycle provides energy for the synthesis of phospholipid substances required by lactic acid bacteria to maintain the stability of their own cell membranes. Glycerol phospholipids also have a competitive utilization relationship for *Clostridium*, which will be decomposed by *Clostridium* to produce acetic acid and butyric acid, accompanied by an increase in NH3–N and pH. In addition to isovaleric acid, there was also an upregulation of isobutyric acid, indicating that the competitive utilization of glycerol–phosphate was the dominant metabolic pathway of *Clostridium*, and the concentration of branched-chain volatile fatty acid (branched VFA) increased. As the moisture content decreased, glycerol–phospholipids were difficult to fully diffuse through the exudate in the silage microenvironment. The pH changes indicated that the competitive intensity of *Clostridium* for nutritional substrates at low moisture is weakened compared with that in 77% and 66% moisture.

### 3.4 Joint analysis of microbial diversity and differential metabolites in silage of *C. fungigraminus*

**FIGURE 5:**
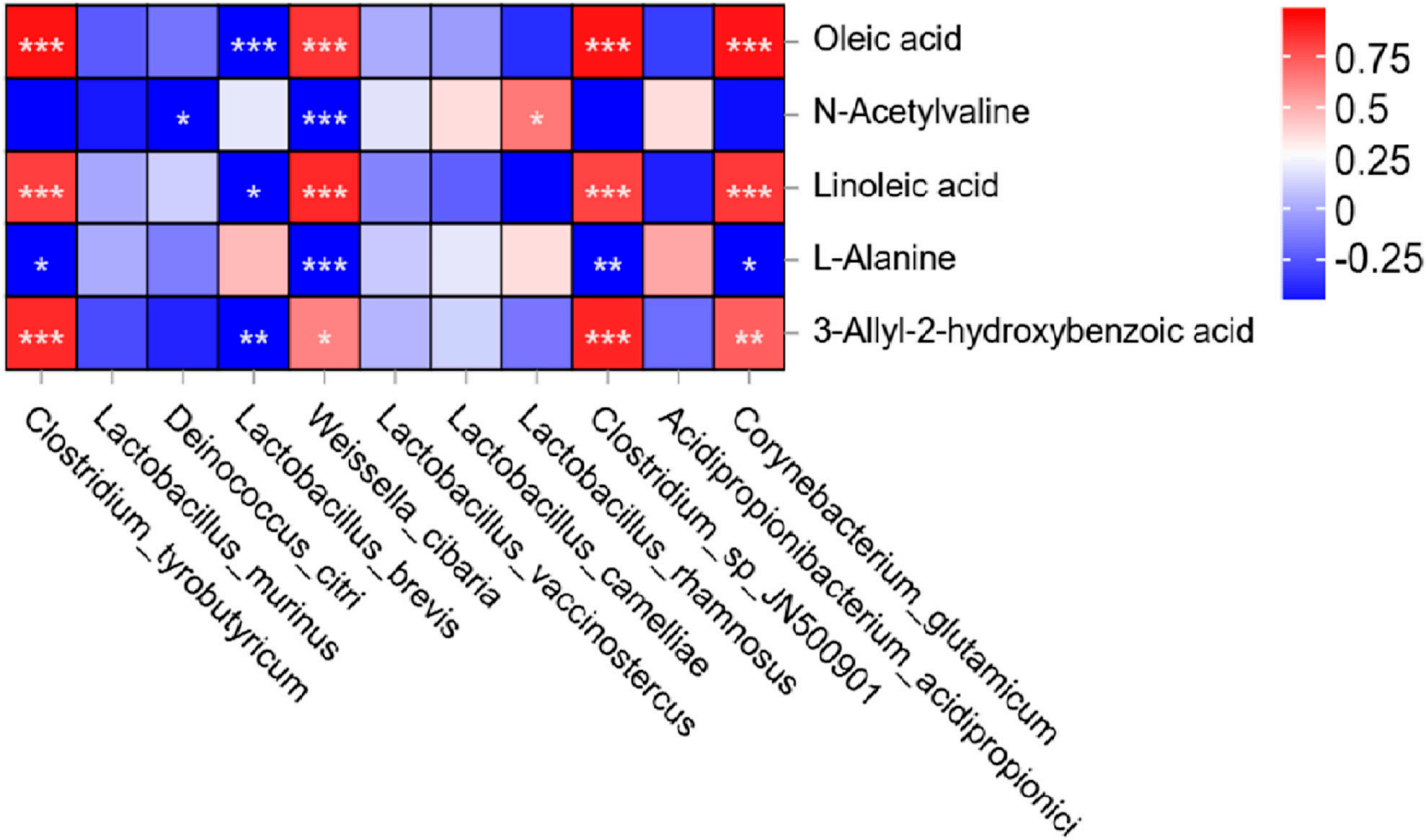
Correlation chart of the relative abundance of key microorganisms and major differential metabolites in silage of *Cenchrus fungigraminus*. Heatmaps show the correlation between species and metabolites between the two groups. The horizontal axis and vertical axis represent the species and metabolites, respectively. The color of the square in the figure indicates the correlation strength and weakness, and the asterisk indicates the correlation significance. One asterisk indicates *p* < 0.05, and two asterisks indicate *p* < 0.01.

This experiment analyzed the association between silage microbial communities and differential metabolites (Figure 5). The stability of oleic acid is closely related to the preparation of high-quality silage. In this experiment, *Clostridium* was significantly correlated with the upregulation of oleic acid and 3-hydroxybenzoic acid (*p* < 0.01), while it was significantly correlated with the downregulation of linoleic acid. *Corynebacterium glutamicum* and *Weissella* were significantly associated with the upregulation of oleic acid. *Clostridium* secretes lipase and phospholipase, which hydrolyze triglycerides, leading to an increase in free oleic acid and a decrease in linoleic acid in silage, both of which are signs of silage corruption. *Corynebacterium glutamicum* provided substrates for the hydrogenation and oxidation of oleic acid and was a harmful microorganism in the silage fermentation process. *Weissella* can counteract the adverse effects of *Clostridium* and *Corynebacterium glutamicum* in the silage microenvironment in various ways. *Lactobacillus brevis* showed a significant negative correlation with the upregulation of oleic acid and 3-hydroxybenzoic acid. 3-hydroxybenzoic acid can penetrate the cell membrane of *Lactobacillus brevis* to block glucose phosphorylation, thus reducing lactic acid/acetic acid production. *Lactobacillus brevis* reduced 3-hydroxybenzoic acid toxicity through decarboxylation modification, but this process slowed the rate of the decrease in pH, extending the proliferation window for *Clostridium*. In this study, *L. brevis* played a dominant role in the silage microbial community, which may explain the poor fermentation quality observed in the 77%, 66%, and 50% moisture treatments. *Weissella* was significantly negatively correlated with the upregulation of valine derivatives (N-acetylvaline). The *Weissella* species detected in this experiment was mainly *Weissella* cibaria, which can convert valine derivatives into acetyl hydroxy acid through homotypic fermentation and was used as a substrate for energy metabolism by ruminants. The downregulation of alanine was significantly negatively correlated with *Weissella* and *Corynebacterium glutamate*. Alanine is a key amino acid for silage modulation. *Weissella cibaraia* can convert alanine into pyruvate, providing a metabolic substrate for lactic acid fermentation and assisting the pH decrease in the silage microenvironment. *Corynebacterium glutamat*e and *Weissella* have a competitive inhibitory relationship, competing for alanine substrate and thereby delaying the efficiency of pH decrease.

## 4 Discussion

### 4.1 Effects of moisture adjustment on the quality characteristics of silage by drying

In this experiment, the CP content of *C. fungigraminus* before silage fermentation was approximately 10%, which is consistent with that in previous studies. Silage fermentation aimed to rapidly reduce the pH to below 4.2, ensuring stable output of high-quality silage (Wang et al., 2017). WSC and DM are considered key factors in reducing pH and inhibiting *Clostridium* (Wilkinson et al., 2003). After drying, the WSC of the raw materials decreased significantly, which may explain why the pH of the silage groups in the drying treatment was higher than that in the 88% moisture group. Generally, the silage microenvironment with low WSC content, high moisture content (>70%), and high pH value (higher than 4.6) is very conducive to the growth of *Clostridium* (Queiroz et al., 2018). Ammonia and amines are produced by converting to butyric acid using WSC, lactic acid, and acetic acid as substrates and dicarboxylic acids. We detected butyric acid in the silage, and the 88% moisture group had the lowest content. This may be because the higher moisture content in the 88% moisture group caused the silage microenvironment to form a weak acid buffer system, which promoted the forward progress of the fermentation of silage feed (Pommel et al., 2006). The LA/AA ratio in the FS group was greater than 2, indicating that homolactic fermentative LAB dominated, but the situation may vary in the other treatment groups. At the same time, the abundance of *Weissella* was also detected in the experiment, which may be one of the reasons why the higher-specific-gravity AA content was detected in moisture groups of 77%, 66%, and 50% in this study (Zhao M. et al., 2022). BA led to the loss of DM and reduced palatability (Muck, 2017). It is an adverse product produced by the fermentation of *Clostridium*. For high-quality forage silage, the concentration of PA should be controlled within 10 g/kg, and the concentration of BA should be controlled within 5 g/kg (Driehuis et al., 2018). Only small amounts of BA and PA were detected in this study, and both were within an acceptable range.

### 4.2 Effects of adjusting moisture on the microbial community of silage in *C. fungigraminus*

The silage of *C. fungigraminus* was mainly *Lactobacillus*, followed by *Weissella*, accompanied by a small amount of *Clostridium*, which was consistent with previous studies (Ni et al., 2019). It showed that exogenous addition of LAB was necessary to rapidly acidify and inhibit *Clostridium* activities and ensure the output of high-quality silage. In this experiment, *Corynebacteriaceae* was detected, which can efficiently produce glutamic acid as a nitrogen source and carbon source for LAB and promote its acid production (Wendisch et al., 2006). Among the dominant genus *Bacillus*, the main bacterial community was *Lactobacillus brevis*, which was consistent with previous studies (Bai et al., 2021). *Weissella* has strong potential in silage fermentation and metabolic engineering, and its relative abundance in silage is second only to *Lactobacillus*. *Weissella* can synthesize A-glucan oligosaccharide, which selectively promote the growth of probiotics such as *Lactobacillus*, inhibit spoilage bacteria through rapid acidification, reduce protein loss, and enhance the nutritional value of feed (Zhang et al., 2020).

### 4.3 Effect of drying and regulating moisture on metabolites of silage in *C. fungigraminus*

This experiment detected changes in phenolic acids. Phenolic acids protect vitamins, pigments, and nutrients in silage by scavenging free radicals, inhibiting lipid oxidation, interfering with metabolic enzyme activity, and suppressing the proliferation of mold, yeast, and other spoilage processes in feed. Succinic acid is an intermediate product of the TCA cycle and can serve as a precursor for microbial metabolism. As the heterotypic fermented LAB detected in this experiment, *Weissella* can generate succinic acid through the reduction branching pathway, which helps regulate pH and inhibits the growth of spoiled bacteria. Butyric acid/isobutyric acid may have a negative impact on the aerobic stability, nutritional value, and palatability of silage. In metabolite detection, it was found that, compared with those in the 88% moisture group, butyric acid metabolites in other treatment groups showed upregulation, and the increase in butyric acid was related to the increase in pH (Kung et al., 2018). This also corresponded to the phenomenon that the pH of the silage in the experimental groups that were dried and treated in this study was higher than that of the 88% moisture group. Protocatechuic acid is a powerful antioxidant that reduces the oxidation reaction caused by plant cell rupture during silage (Meyer et al., 1998). This improves the aerobic stability of the silage. Protocatechuic acid can inhibit protein synthesis of *E. coli* and *Clostridium*, reduce the accumulation of NH_3_–N in silage, and reduce the risk of spoilage (Lu et al., 2012).

This experiment detected a significant change in amino acids. Amino acids play a very important role in promoting the reproduction of LAB, enhancing fermentation efficiency, and indirectly promoting the generation of LAB. They are key factors in regulating fermentation dynamics. Aspartic acid serves as a nitrogen source for beneficial bacteria such as LAB, participates in the urea cycle, promotes fermentation efficiency, eliminates free radicals, slows degradation of silage, and helps maintain nutrient stability. The content of aspartic acid in alfalfa silage was positively correlated with the milk protein yield in dairy cows (Kim et al., 1999). Glutamate, as a neurotransmitter precursor, may have an impact on the appetite and digestive function of livestock. Studies have shown that glutamate can increase the intestinal digestive function of broiler chickens, thereby improving digestibility (Huang et al., 2010). The content of lysine in silage can directly affect the growth performance of livestock. For ruminants, the balance of lysine and methionine is crucial for milk protein synthesis (Galo et al., 2003). In this experiment, the equilibrium relationship of the above amino acids changed, probably reducing protein utilization.

## 5 Conclusion

Adjusting the moisture content of raw *C. fungigraminus* through drying had dual effects on fresh forage quality. As drying time increased, microbial richness and diversity in the raw material increased, while WSC and EE levels decreased, and ADF content also decreased. At lower moisture levels, available substrates for silage fermentation were reduced, thereby altering the fermentation dynamics. This study emphasized the interconnected effects of moisture content, microbial community composition, and metabolite profiles on silage fermentation quality within the dynamic microenvironment of *C. fungigraminus* silage. Our results indicate that *C. fungigraminus* may have greater silage potential under higher moisture conditions as >75% moisture content was more favorable for LAB dominance, fermentation stability, and nutritional preservation.

## Data Availability

The data presented in the study are deposited in the Genome Sequence Archive of the Big Data Centre at the Beijing Institute of Genomics (BIG), Chinese Academy of Sciences (http://bigd.big.ac.cn), accession number CRA027467.
